# Association between dietary patterns and sleep quality in Chinese children and adolescents: large-scale cross-sectional network analysis

**DOI:** 10.3389/fnut.2026.1851693

**Published:** 2026-07-01

**Authors:** Yi Yang, Duo Liu, Yayu Yang, Junhua Wang, Lina Xu, Zhujun Qiao, Yongbo Zou, Zhongquan Chen, Yuandong Hu

**Affiliations:** 1 School of Public Health, The Key Laboratory of Environmental Pollution Monitoring and Disease Control, Ministry of Education, Guizhou Medical University, Guiyang, Guizhou, China; 2 Guizhou Center for Disease Control and Prevention, Guiyang, Guizhou, China; 3 Renhuai Maternal and Child Health Center, Zunyi, Guizhou, China; 4 Renhuai Center for Disease Control and Prevention, Zunyi, Guizhou, China

**Keywords:** child health, dietary pattern, network analysis, principal component analysis, sleep quality

## Abstract

**Background:**

With shifts in lifestyle and living environments, the overall health of children and adolescents presents cause for concern, with sleep disorders becoming increasingly prevalent. Diet, as a modifiable lifestyle factor, significantly in-fluences sleep quality.

**Methods:**

A cross-sectional study was conducted in Renhuai City, Guizhou Province, China. A food-frequency survey was conducted among 13,523 children and adolescents, and dietary patterns were extracted using principal component analysis. Stratified by age (children and adolescents), logistic regression was used to examine the association between sleep quality and dietary patterns. Network analysis was used to explore the relationships between dietary patterns and various sleep dimensions in children and adolescents, identify bridge symptoms, and compare network characteristics between the two groups.

**Results:**

Four dietary patterns were identified High-Protein, Plant-Based, Fast Food and Snack, and Nuts-Aquatic products -Potatoes. Regression analyses in both children and adolescents identified the Fast Food and Snack pattern as the highest-risk pattern for sleep quality. Network analysis showed that in children, the Fast Food and Snack–Sleep Disturbance edge had the highest strength, and this diet pattern also showed the greatest bridge strength. In adolescents, the strongest edge was between the Nuts-Aquatic products -Potatoes Diet Type -Daytime function, this diet pattern also had the highest bridge strength. The global network structure differed significantly across age groups.

**Discussion:**

The association between diet patterns and sleep quality differs between children and adolescents. For children, the Fast Food and Snack Diet Type is associated with poorer sleep condition; whereas for adolescents, the Nuts-Aquatic products -Potatoes Diet Type is associated with good sleep quality.

## 1 Introduction

With changes in lifestyle and living environments, the overall health of children and adolescents is not promising (1). Sleep, a crucial physiological process for growth and development, is vital for the healthy development of children and adolescents (2). In Asia, approximately 51.9% of children suffer from sleep disorders (3). In China, the prevalence of sleep problems among children has also been on the rise in recent years, affecting two-fifths of school-aged children (4). Furthermore, after entering adolescence, sleep problems will become even more severe, and teenagers will encounter more sleep issues (5). Studies have shown that sleep disorders are closely related to various dis-eases in children and adolescents, including obesity (6), myopia (7), and cognitive and psychological disorders (8, 9). Therefore, early identification of the risk factors for sleep problems and the implementation of targeted interventions are of great significance for promoting the health of this group.

The sleep of children and adolescents is influenced by multiple factors, including prolonged use of electronic devices (10), excessive homework, emotional disorders (11), and physical activities (12). Among them, diet, as a modifiable lifestyle factor, has a significant impact on sleep quality (13). Unhealthy eating habits not only affect day-time alertness but also influence nighttime sleep (14). If children and adolescents have a higher compliance with the Mediterranean diet, their sleep quality will be better (15); while if they consume more ultra-processed foods (UPF), their sleep quality will be worse (16). In research on dietary patterns, “vegetables and healthy proteins”, “traditional diet”, and “fruits and vegetables” are considered beneficial for sleep (17).

Sleep is a multi-dimensional concept. Besides the simple duration and quality of sleep, it is also important to consider daytime functioning and sleep disorders. However, existing studies are limited to investigating the impact of a single dietary behavior on sleep (18) or only exploring the influence of certain dietary patterns on sleep from an overall perspective (19), and have not delved deeply into the relationship be-tween dietary patterns and sleep microstructure. Traditional regression analysis is limited to focusing only on isolated associations between predictors and outcomes. As an emerging statistical method, network analysis (NA) overcomes this limitation by converting multiple variables into nodes in a network and intuitively visualizing the complex interrelationships among variables through the connections between nodes, thereby allowing for the exploration of deeper associations at the symptom level (20). Furthermore, according to the centrality theory of the network, the most influential symptoms are regarded as central nodes, while bridge symptoms connect different symptom clusters (21) and serve as important nodes and bridges that break down the symptom network. The application of NA can be used to identify central nodes and bridge symptoms between dietary patterns and sleep, and to identify key breakthrough points for the overall dietary sleep intervention. Children and adolescents, as a group with diverse develop-mental characteristics, exhibit significant differences in behavioral lifestyles such as diet and sleep (5, 22). Therefore, in this study, we stratified by age (children and adolescents) and comprehensively employed traditional regression models and network analysis methods. We systematically explored the impact of dietary patterns on sleep from both macro-level correlation and micro-level structure perspectives, providing a basis for future intervention and prevention efforts to improve sleep health in children and adolescents.

## 2 Materials and methods

### 2.1 Research subjects

**Figure 1:**
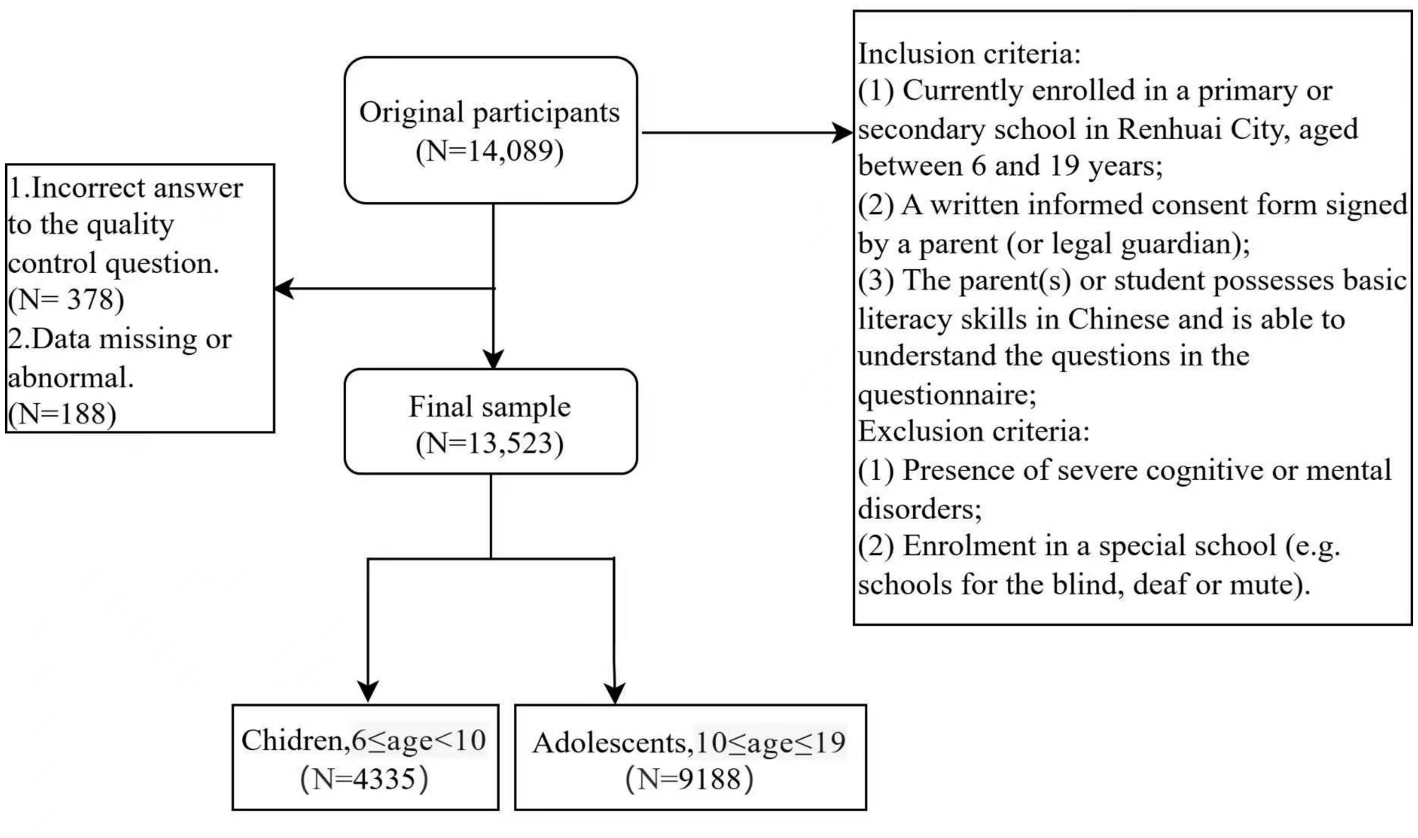
Flow chart of participant inclusion and exclusion.

This study used non-random sampling and was completed through online recruitment. This cross-sectional study was conducted in Renhuai City, Guizhou Province, China, from November to December 2025. The QR code for the online questionnaire was distributed by homeroom teachers to class parent WeChat groups. A total of 14,089 parents, their children, and adolescents agreed to participate and completed the online questionnaire. The questionnaire included one quality-control question; those who answered incorrectly were deemed invalid, and 378 questionnaires were excluded, yielding an effective rate of 97.3%. Additionally, 188 questionnaires with missing or abnormal data were removed. Finally, 13,523 valid questionnaires were obtained. The inclusion and exclusion flow chart is shown in Figure 1. This research plan was reviewed and approved by the Ethics Committee of the Guizhou Provincial Center for Disease Control and Prevention (Approval Number: Q2025-15), and all participants provided informed consent.

### 2.2 Method

#### 2.2.1 Questionnaire

This study collected information through the Questionstar online questionnaire, including the following aspects:

Demographic factors: gender, age, smoking status, drinking status, and parental marital status.

Family socioeconomic status (SES): father’s education level, mother’s education level, father’s occupation type, mother’s occupation type, and annual household in-come.

Education level was classified as: primary school or below, junior high school, senior high school or technical secondary school, college (bachelor’s or associate degree), graduate school or above (23).

Occupation type was classified as: state and social managers, managerial personnel, private entrepreneurs, professionals and technicians, clerical staff, self-employed individuals, commercial and service workers, industrial workers, agricultural laborers, and the unemployed, semi-unemployed, or jobless in urban and rural areas (24).

Annual household income was classified as: less than 30,000 ¥, 30,000–50,000 ¥, 50,000–100,000 ¥, 100,000–150,000 ¥, 150,000–200,000 ¥, 200,000–250,000 ¥, or above 250,000 ¥ (25).

Physical activity: Moderate-to-vigorous physical activity (MVPA) duration was assessed using the Evaluation of Physical Activity Levels in Children and Adolescents (WS/T 10008—2023). A daily duration of ≥ 70 min indicated that physical activity met the standard, whereas < 70 min indicated that physical activity was below the standard (26).

Screen time: Screen time was assessed using the Questionnaire on Static Behavior in Children and Adolescents (WS/T 10008—2023). A daily screen time of ≤ 2 h indicated that screen usage met the standard, whereas > 2 h indicated that it was below the standard (26).

Caffeine intake: Within the past month, the intake frequency per week of coffee frequency options were never, 1–2 days/week, 3–4 days/week, 5–6 days/week, and every day, assigned scores of 0, 1, 2, 3, and 4 respectively, with higher scores indicating higher coffee intake.

Staying up late: Falling asleep after 11 p.m. is defined as staying up late (27).

Food diversity: Within the past month, the intake frequency per week of dairy products, soybeans and soybean products, nuts, livestock meat and its products, poultry meat and its products, aquatic products, eggs, vegetables, grains, fruits, potatoes and tubers, snacks, fried foods, and sugar-sweetened beverages (Cronbach’s *α* = 0.805, Validity = 0.847) (28, 29).

Sleep quality: measured using the Pittsburgh Sleep Quality Index (PSQI) (Cronbach’s *α* = 0.719) (30). Each component is scored on a scale of 0 to 3: subjective sleep quality, sleep efficiency, sleep duration, sleep latency, daytime function, drug use, and sleep disturbance. The sum of these scores yields a global PSQI score ranging from 0 to 21, with higher scores indicating poorer sleep quality. Sleep quality was categorized as good when the PSQI score was ≤ 5 (PSQI ≤ 5), and poor when the PSQI score was > 5 (PSQI > 5) (31, 32).

#### 2.2.2 Covariate

Include the following factors: gender, age, SES, parental marital status, smoking status, drinking status, physical activity, screen time, caffeine intake and staying up late.

#### 2.2.3 Statistical analysis

SES used SPSS Professional 31.0 for principal component analysis (PCA). The calculation was based on family income level, parents’ educational attainment, and occupation type. The income level was divided into 7 grades, ranging from the lowest “1 = less than 30,000 ¥” to the highest “7 = more than 250,000 ¥”. The educational level was calculated as the highest educational attainment of either parent and divided into 5 grades, ranging from the lowest “1 = primary school or below” to the highest “5 = post-graduate or above”. The occupation was calculated based on the highest occupation level of either parent and was divided into 10 grades, ranging from the lowest “1 = urban rural unemployed, semi-unemployed, or self-employed” to the highest “10 = national and social managers” (33). Subsequently, based on the results of the KMO test and Bartlett’s sphericity test, it was determined whether principal component analysis was suitable, and the factor integration was calculated.

The dietary pattern used SPSS Professional Version 31.0 for PCA. The frequency of food consumption was scored based on food diversity and divided into 5 evaluation levels: never, 1–2 days/week, 3–4 days/week, 5–6 days/week, and every day. Each level was assigned the following scores: 0, 1, 2, 3, and 4 points. Subsequently, based on the results of the KMO test and Bartlett’s sphericity test, it was determined whether principal component analysis was suitable, and the factor integration was calculated.

SPSS Professional Version 31.0 was used to conduct chi-square tests, rank-sum tests, and multivariate logistic regression to explore the association between sleep quality and dietary patterns.

Network analysis was performed using the qgraph package in R 4.3.3 to visualize the network model of dietary patterns and sleep dimensions. First, we standardized the variables using a z-score transformation to ensure comparability. For the overall network, edges represent the partial correlation between each pair of nodes after controlling for all other nodes. Given the non-normality of the variables, we estimated the network using Spearman’s partial correlations. We also applied the least absolute shrinkage and selection operator (LASSO) with the extended Bayesian information criterion (EBIC) and set the tuning parameter to its default value of 0.5 (34). This approach shrinks weak correlations, resulting in a sparser, more stable, and clearer network (35). The “networktools “package was used to calculate bridge centrality indices and bridge strength to identify bridge symptoms within the network model. The “Network Comparison Test” package was employed to test for in-variance in both network structure and global strength, while edge weight invariance was used to evaluate local differences. The “bootnet” package was used to assess the accuracy of edge-weight estimation and the stability of centrality indices. Nonparametric bootstrap methods were applied to estimate the 95% confidence intervals (CIs) for edge weights, with narrower CIs indicating lower network volatility. The stability of centrality indices was evaluated using the correlation stability coefficient (CS). A CS value greater than 0.25 indicates minimum stability; greater than 0.5 indicates sufficient stability; and greater than 0.70 indicates high stability (36, 37).

## 3 Results

### 3.1 SES results for children and adolescents

After assigning scores to family income level, parents’ educational attainment, and occupation type and standardizing the data, the results showed that *KMO* = 0.685 and Bartlett’s sphericity test *χ*^2^ = 9110.736, *p* < 0.001. Based on the criterion of eigenvalues greater than 1, only one principal component was extracted (the characteristic root = 1.966), which explained 65.538% of the total variance. For the first principal component, the factor loadings for family income level, educational attainment, and occupation type were 0.794, 0.812, and 0.822, respectively. That is, in this study, SES = (0.794 × *Z* family annual income + 0.812 × *Z* educational attainment + 0.822 × *Z* occupation type)/1.966.

### 3.2 Dietary patterns results among children and adolescents

After assigning scores to the frequency of consumption of various foods and standardizing the data, the results showed that KMO = 0.851, Bartlett’s sphericity test *χ*^2^ = 47356.733,*p* < 0.001, to achieve clearer naming, the variance-maximizing orthogonal rotation method was used to rotate the factor component matrix. After rotation, 4 factors had eigenvalues greater than 1: 2.250, 2.029, 1.905, and 1.888, accounting for 57.667% of the total variance. The rotated factors with loadings greater than 0.5 were selected as representatives for type naming (38). The name given to each pattern is based on understanding the variable content and the respective characteristics of each dietary pattern. The factor model of the 4 dietary patterns was used to obtain dietary pattern factor scores, and the maximum score was used to classify each individual’s dietary pattern. The factor loadings are shown in Table 1.

**Table 1:** Factor loadings of dietary patterns for children and adolescents.

Food	High-protein diet type	Plant-based diet type	Fast food and snack diet type	Nuts-aquatic products-potatoes diet type
Dairy products	0.705			
Soybeans and soybean	0.719			
Livestock meat and its products	0.628			
Poultry meat and its products	0.637			
Eggs				
Nuts				0.648
Aquatic products				0.628
Fruits		0.646		
Vegetables		0.763		
Potatoes and tubers				0.774
Grains		0.733		
Snacks			0.770	
Fried foods			0.781	
Sugar-sweetened beverages			0.763	

The eggs did not reach the required weight, so they are not included.

### 3.3 Single-factor analysis of sleep quality among children and adolescents

The chi-square test and rank sum test showed that in children, drinking status, smoking status, parental marital status, SES, age, staying up late, caffeine intake, and dietary pattern had significant effects on sleep quality (*p* < 0.05); in adolescents, gender, drinking status, smoking status, parental marital status, age, staying up late, physical activity, screen time, caffeine intake, and dietary pattern also had significant effects on sleep quality (*p* < 0.05). The results of the single-factor analysis are shown in Table 2.

**Table 2:** Single-factor analysis of sleep quality of children and adolescents.

Characteristics	Children (*n* = 4,335)				Adolescents (*n* = 9,188)			
	Poor sleep quality (%)	Good sleep quality (%)	*χ*^2^/Z	*p*-value	Poor sleep quality (%)	Good sleep quality (%)	*χ*^2^/Z	*p*-value
Gender			0.912	0.34			59.068	0.001 ***
Male	112(4.8)	2,210 (95.2)			663 (15.1)	3,741 (84.9)		
Female	110 (5.5)	1903 (94.5)			1,017 (21.3)	3,767 (78.7)		
Drinking			30.797	0.001 ***			125.53	0.001***
No	213 (5.0)	4,087 (95.0)			1,527 (17.3)	7,278 (82.7)		
Yes	9 (25.7)	26 (74.3)			153 (39.9)	230 (60.1)		
Smoking			20.918	0.001 ***			58.99	0.001 ***
No	216 (5.0)	4,096 (95.0)			1,585 (17.8)	7,342 (82.2)		
Yes	6 (26.1)	17 (73.9)			95 (36.4)	166 (63.6)		
Parental marital status			8.54	0.036*			15.121	0.002**
Normal	186 (4.8)	3,667 (95.2)			1,300 (17.5)	6,118 (82.5)		
Divorced	27 (6.8)	372 (93.2)			291 (21.2)	1,080 (78.8)		
One or both parents deceased	2 (10.0)	18 (90.0)			41 (22.4)	142 (77.6)		
Unclear	7 (11.1)	56 (88.9)			48 (22.2)	168 (77.8)		
SES	5.4 (4.0,8.1)	6.7 (4.3,9.9)	−4.079	0.001 ***	5.1 (4.0,7.3)	5.1 (4.0,7.3)	−0.118	0.906
M (P25, P75)								
Age	8 (7,9)	8 (7,8)	−3.188	0.001 ***	16 (13,17)	13 (11,15)	−27.05	0.001***
M (P25, P75)								
Staying up late			50.109	0.001 ***			979.14	0.001***
No	199 (4.7)	4,016 (95.3)			746 (10.9)	6,098 (89.1)		
Yes	23 (19.2)	97 (80.8)			934 (39.8)	1,410 (60.2)		
Physical activity			1.213	0.271			50.589	0.001***
Not meeting	84 (4.7)	1710 (95.3)			739 (22.1)	2,609 (77.9)		
Meeting	138 (5.4)	2,403 (94.6)			941 (16.1)	4,899 (83.9)		
Screen time			0.238	0.626			39.229	0.001 ***
Not meeting	7 (4.3)	156 (95.7)			177 (27.5)	467 (72.5)		
Meeting	215 (5.2)	3,957 (94.8)			1,503 (17.6)	7,041 (82.4)		
Caffeine intake M (P25, P75)	0 (0,0)	0 (0,0)	4.183	0.001 ***	0 (0,1)	0 (0,0)	16.407	0.001 ***
Dietary pattern			53.78	0.001 ***			229.86	0.001***
Nuts-aquatic products -potatoes	74 (6.5)	1,070 (93.5)			345 (14.5)	2028 (85.5)		
Plant-based	35 (2.6)	1,291 (97.4)			306 (12.4)	2,164 (87.6)		
High-protein	28 (3.2)	859 (96.8)			538 (24.0)	1700 (76.0)		
Fast food and snack	85 (8.7)	893 (91.3)			491 (23.3)	1,616 (76.7)		

***p < 0.001; **p < 0.01; *p < 0.05.

### 3.4 Multivariate logistic regression analysis of sleep quality of children and adolescents

The results of the multiple logistic regression analysis for both children and adolescents showed that, after controlling for gender, drinking status, smoking status, parental marital status, SES, age, staying up late, physical activity, screen time, and caffeine intake, compared with the Fast Food and Snack dietary pattern, the Nuts-Aquatic products-Potatoes pattern, the Plant-Based pattern, and the High-Protein pattern were all protective factors for sleep quality (*p* < 0.05). The results of the multiple logistic regression analysis are shown in Table 3.

**Table 3:** Multivariate logistic regression analysis of sleep quality of children and adolescents.

Variables		Children		Adolescents	
		OR (95% CI)	*p*	OR (95% CI)	*p*
Dietary pattern	Fast food and snack	1.00 (ref.)	–	1.00 (ref.)	–
	High-protein	0.415 (0.265,0.650)	<0.001***	0.825 (0.706,0.965)	0.016*
	Nuts-aquatic products -potatoes	0.690 (0.495,0.962)	0.029*	0.629 (0.533,0.742)	< 0.001***
	Plant-based	0.307 (0.204,0.463)	<0.001***	0.579 (0.489,0.687)	< 0.001***

Taking good sleep quality as the reference, the odds ratio (OR) and 95% confidence interval (CI) for poor sleep quality are shown in this Table. ***p < 0.001; **p < 0.01; *p < 0.05. Model: adjusted for variables age, SES, gender, Parental marital status, smoking, drinking, Coffee intake, staying up late, physical activity status, screen time status.

### 3.5 Network analysis of sleep quality and dietary patterns among children and adolescents

#### 3.5.1 Network of children’s sleep quality and dietary patterns

**Figure 2:**
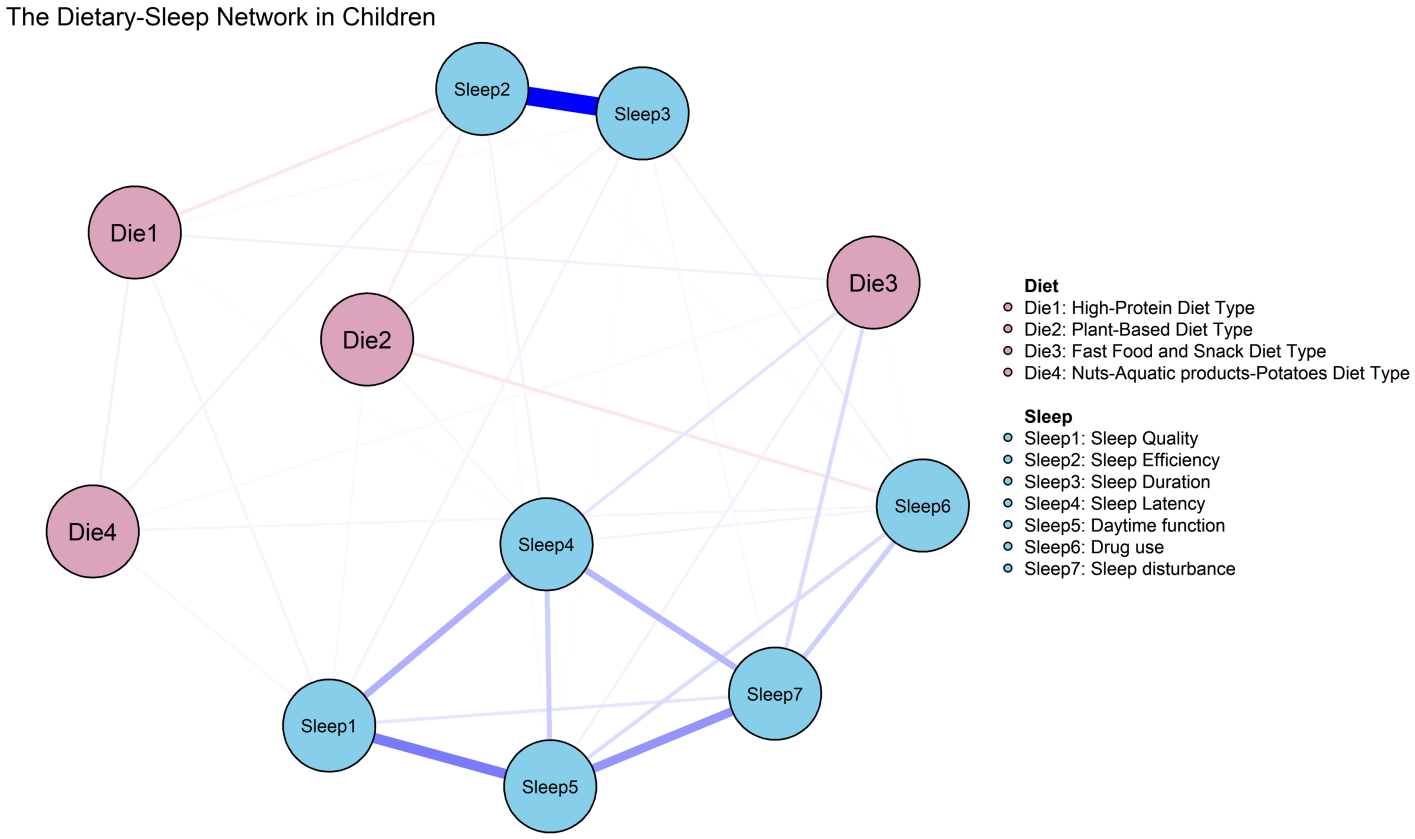
Network of children’s sleep quality and dietary pattern.

In the children’s diet and sleep network, the network density was 0.673 (37/55), and the average edge weight was 0.035. The 11 nodes generated 55 potential edges, among which 37 edges had non-zero weights. For children’s sleep quality and dietary patterns, the strongest partial correlation was between the Fast Food and Snack Diet type and sleep disturbance (edge weight = 0.077).as shown in Figure 2. The network weight matrix table is provided in Supplementary Table S1.

#### 3.5.2 Network of adolescent sleep quality and dietary patterns

**Figure 3:**
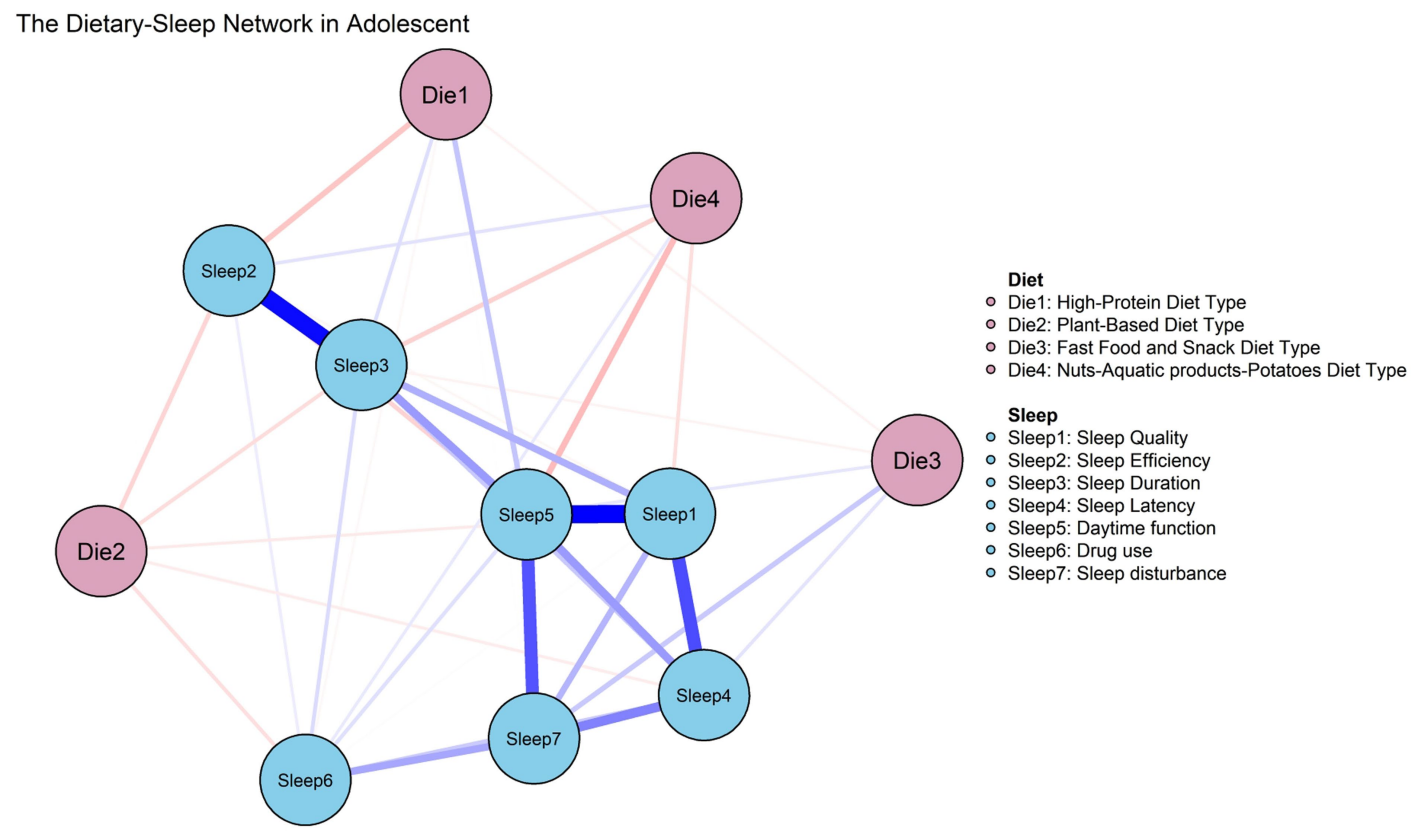
Network of adolescent sleep quality and dietary pattern.

In the adolescent diet and sleep network, the network density was 0.690 (38/55), and the average edge weight was 0.037. The 11 nodes generated 55 potential edges, among which 38 edges had non-zero weights. The strongest partial correlation between adolescents’ sleep quality and the dietary pattern network was observed for the Nuts-Aquatic products -Potatoes Diet Type - Daytime function (edge weight = −0.098), as shown in Figure 3. The network weight matrix table is provided in Supplementary Table S2.

#### 3.5.3 Bridge symptoms between children’s sleep quality and dietary patterns

**Figure 4:**
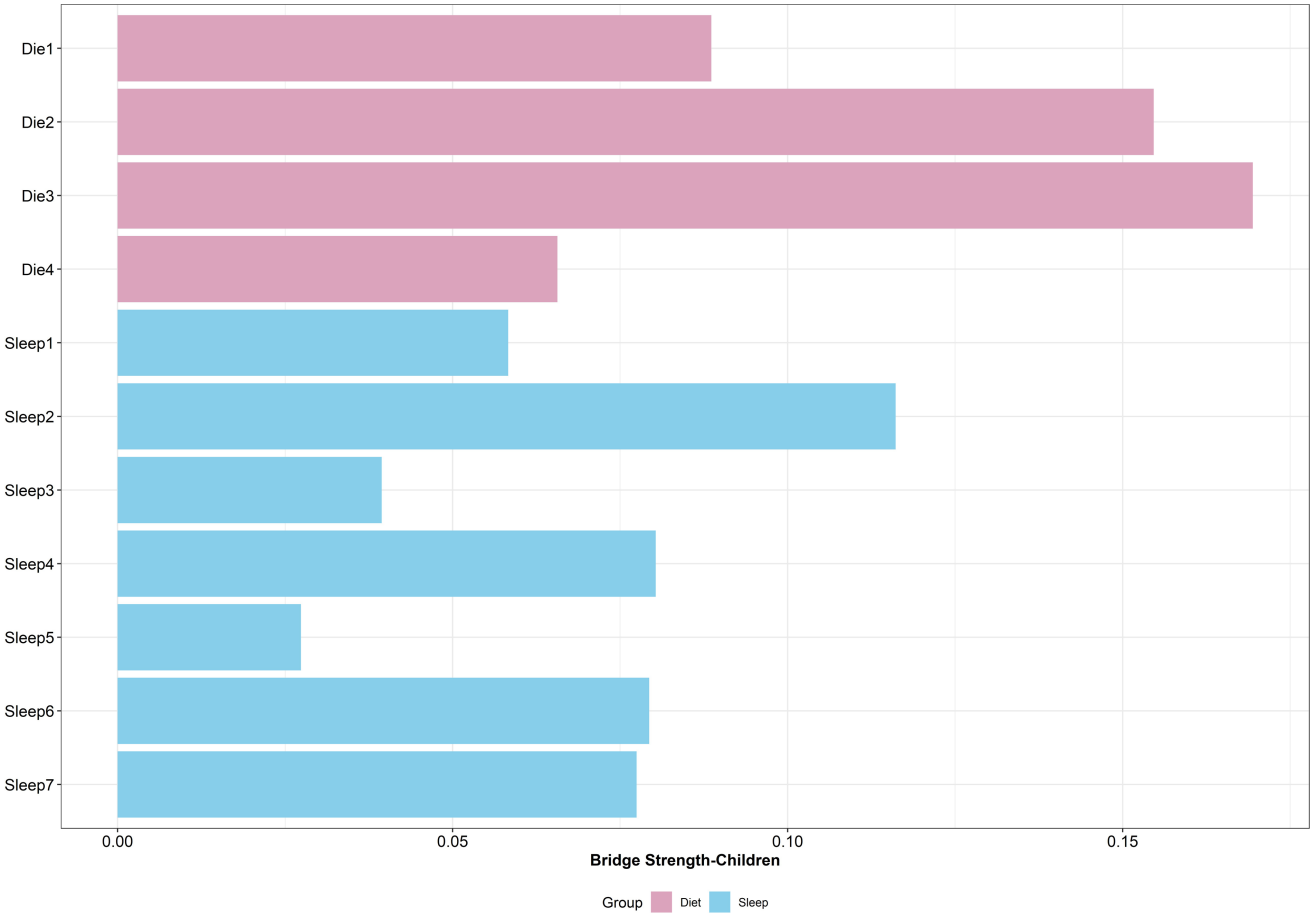
Bridge symptoms in the network of children’s sleep quality and dietary patterns.

In the network analysis of children’s diet and sleep, bridge centrality results indicated that, within the dietary patterns dimension, the Fast Food and Snack Dietary Type had the highest bridge strength, while, within the sleep dimension, Sleep Efficiency had the greatest bridge strength, as shown in Figure 4.

#### 3.5.4 Bridge symptoms between adolescent sleep quality and dietary patterns

**Figure 5:**
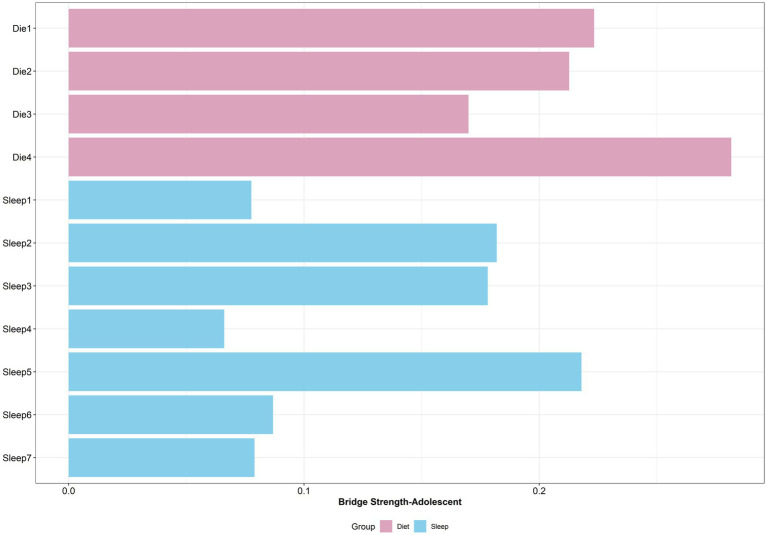
Bridge symptoms in the network of adolescent sleep quality and dietary patterns.

In the network analysis of adolescent diet and sleep, bridge centrality results indicated that within the dietary patterns dimension, the Nuts-Aquatic products -Potatoes Diet Type demonstrated the highest bridge strength, while in the sleep dimension, Daytime function exhibited the greatest bridge strength, as shown in Figure 5.

#### 3.5.5 Stability of network and bridge symptoms

**Figure 6:**
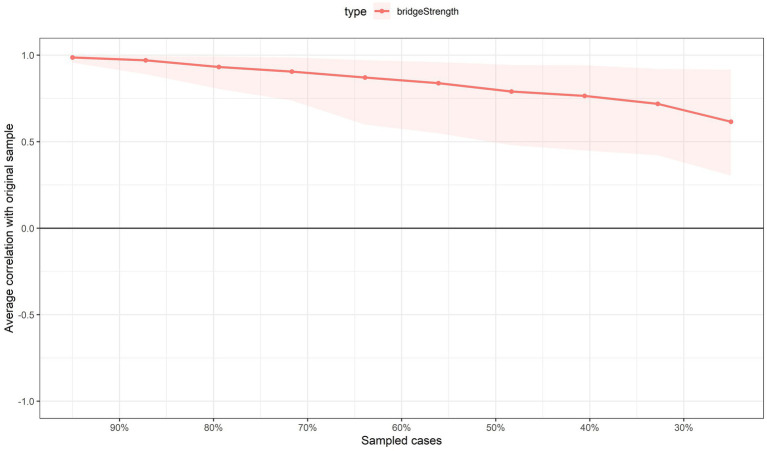
Stability of bridge strength in children.

In the children’s diet-sleep network and bridge symptom analysis, Supplementary Figure S1. indicates that the bootstrapped 95% confidence intervals for edge weights are relatively narrow, suggesting acceptable reliability of the network model. Supplementary Figures S2, S3 present the results of nonparametric bootstrap difference tests for edge weights and node strength, respectively. Most edges and nodes were significantly different from one another, supporting the credibility of the main findings. For bridge symptoms in children, the correlation stability coefficient (CS) for bridge strength was 0.361as shown in Figure 6.

**Figure 7:**
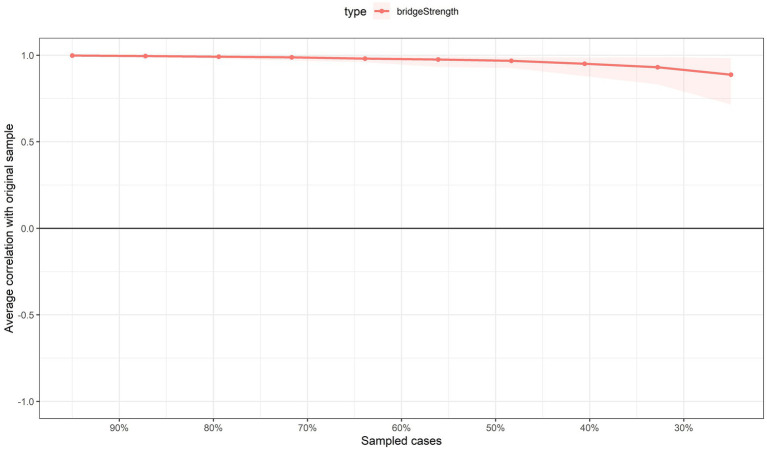
Stability of bridge strength in adolescent.

In the analysis of bridge strength within the adolescent diet-sleep network, Supplementary Figure S4 shows that the bootstrapped 95% confidence intervals for edge weights are relatively narrow, indicating acceptable reliability of the network model. Supplementary Figures S5, S6 present the results of nonparametric bootstrap difference tests for edge weights and node strength, respectively. Most edges and nodes were significantly different from one another, supporting the credibility of the main findings. For bridge symptoms in adolescents, the correlation stability coefficient (CS) for bridge strength was 0.75as shown in Figure 7.

#### 3.5.6 Comparison of network characteristics between children and adolescents

There were age-related differences in global network strength: adolescents = 3.291, children = 2.469 (*p* < 0.001). Permutation-based network comparison tests with 1,000 permutations further indicated significant differences in both the overall network structure (*M* = 0.218, *p* = 0.001) and global connection strength (S = 0.821, *p* = 0.001) be-tween the two groups. In the network between sleep and dietary patterns, the following edge pairs showed significant differences: Nuts-Aquatic products -Potatoes Diet Type – Daytime function; Nuts-Aquatic products -Potatoes Diet Type – Sleep Duration; High-Protein Dietary Type– Daytime function; High-Protein Dietary Type – Sleep Duration; and High-Protein Dietary Pattern – Sleep Quality. Moreover, these connections were consistently stronger in adolescents than in children, as shown in Supplementary Table S3.

## 4 Discussion

This study extracted four dietary patterns through PCA: High-Protein Diet Type (HPD), Plant-Based Diet Type (PBD), Fast Food and Snack Diet Type (FFSD), and Nuts-Aquatic products -Potatoes Diet Type (NAP). The four dietary patterns were all significantly associated with sleep quality in the single-factor analysis of the child and adolescent groups. In the multi-factor analysis, after controlling for gender, age, SES, parental marital status, smoking status, drinking status, physical activity, screen time, caffeine intake and staying up late, the multi-factor analysis results for the child group showed that when FFSD was set as the reference group, the HPD and PBD, and NAP were all protective factors for sleep quality; the analysis results for the adolescent group were consistent with those for the child group. In the network analysis, among the relationships between children’s sleep quality and dietary patterns, FFSD - Sleep Disturbance (edge weight = 0.077) had the highest intensity, and the bridging strength of FFSD was the highest; Among the relationships between the sleep quality of adolescent and dietary patterns, the NAP - Daytime function (edge weight = −0.098) had the highest intensity, and the bridging strength of NAP was the highest. Furthermore, there is a significant age disparity worldwide on the internet. These results provide a theoretical basis for im-proving the sleep quality of children and adolescents through dietary patterns.

In the multi-factor analysis, across both children and adolescents, compared with the FFSD, the NAP, PBD, and HPD all demonstrated protective effects on sleep. This suggests that the FFSD might be the most dangerous dietary pattern affecting sleep quality. The FFSD mainly consists of snacks, sugary beverages, and fried foods. These foods are mostly classified as UPF. UPF is believed to significantly affect sleep (16, 39), which is consistent with this study. With the intake of UPF, the risks of insomnia (40), shortened sleep duration (39), and poor sleep quality (41) also increase accordingly. The mechanism may lie in the fact that intake of UPF leads to destruction of the intestinal microbiota (42), and the composition of the intestinal microbiota is believed to be associated with insomnia (43), thereby possibly causing a series of sleep problems. Furthermore, it has been confirmed that sleep problems can also affect the secretion of appetite-regulating hormones, such as ghrelin and leptin. This may lead to a vicious cycle between sleep and eating disorders (44).

In the network analysis, the strongest partial correlation between the network of children’s sleep quality and dietary patterns was FFSD - Sleep Disturbance (edge weight = 0.077). The FFSD is associated with sleep disturbances, consistent with previous studies (45–47). The underlying physiological mechanism is that children’s digestive systems are not yet fully developed, while the FFSD has a low fiber content but high levels of saturated fat, salt, and sugar (48). These components are not only associated with decreased sleep quality and reduced slow-wave sleep on the whole (49), but also because they are difficult for children to digest, they may cause irritation to the digestive system, leading to functional disorders of the gastrointestinal system and causing digestive tract symptoms such as abdominal distension, abdominal pain, diarrhea, etc. (50). These symptoms result in sleep disorders and prolonged latency in children, thereby affecting sleep quality (51). Among adolescents, the strongest partial correlation was found for the NAP - Daytime Function (edge weight = −0.098), indicating that the NAP is associated with a better daytime function. Studies have shown that adolescents face heavy academic burdens (52) and undergo greater physiological changes, which can lead to increased sleep problems (5). The NAP is characterized by rich contents of complex carbohydrates, dietary fiber, unsaturated fatty acids, tryptophan, and protein (53–55), and is crucial for the initiation and maintenance of sleep (56, 57). These nutrients do not act independently; they work synergistically. Tryptophan serves as the precursor for the synthesis of serotonin and melatonin, serving as the biochemical basis for directly initiating sleep signals and shortening the sleep latency (58); Unsaturated fatty acids, through their an-ti-inflammatory and neurotrophic properties, help maintain the stability and health of the nervous system, potentially promoting slow-wave sleep, thereby optimizing the sleep structure (59); Dietary fiber and complex carbohydrates, by delaying glucose ab-sorption, jointly maintain the nighttime blood sugar stability and prevent nocturnal awakenings due to energy fluctuations, effectively enhancing sleep efficiency and continuity (60). Ultimately, this promotes improvements in overall sleep quality and enhances daytime functioning in adolescents.

The bridge symptoms in the dietary pattern may serve as a key target for improving sleep quality, as interventions targeting these symptoms may alter the corresponding sleep symptom clusters. Among children, the bridging symptom in the dietary pattern is FFSD; among adolescents, it is NAP. Therefore, different intervention methods should be adopted for different age groups. For children, the intervention should focus on avoiding FFSD, while for adolescents, NAP should be adopted.

There are age-related differences in the network of sleep quality and dietary pat-terns. The network structure of adolescents is denser than that of children, indicating stronger connections between sleep quality and dietary patterns. This difference may be attributed to teenagers having greater autonomy in food choices, being more frequently involved in social activities, being exposed to electronic screens, and facing academic pressure (52). These factors not only directly affect sleep but also lead to a more diverse diet pattern. From a physiological perspective, adolescents also experience sleep problems due to the delayed phase of circadian rhythms, which is caused by changes in sex hormones during puberty (61). Furthermore, the edge strengths for NAP - Daytime function, NAP- Sleep Duration, HPD - Daytime function, HPD - Sleep Duration, and HPD - Sleep Quality were stronger in adolescents than in children. A possible reason is that adolescents have greater exposure to online media than children, and online media can increase adolescents’ attention to body management (62), leading them to choose the NAP and HPD more often, thereby strengthening the connection with sleep.

This study also has certain limitations. 1. As a cross-sectional study, we cannot establish causal relationships. The network analysis performed in this study is also limited in its causal interpretation and should be considered exploratory. Future longitudinal studies are needed to support causal inferences. 2. The survey was conducted in a single city, which may limit the generalizability of our findings to other populations or regions. 3. The dietary assessment only investigated food intake frequency and did not quantify portion sizes, potentially affecting the accuracy of dietary exposure estimation. 4. Despite adjusting for multiple covariates, the possibility of residual confounding due to unmeasured or inadequately measured factors cannot be ruled out. 5. This study used non-random sampling, which may introduce selection bias and limit the representativeness of the sample. In summary, this study combines traditional regression analysis with network analysis methods to comprehensively explore the correlation between children’s and adolescents’ sleep quality and dietary patterns at both macro- and micro-levels. It has identified key connections between sleep and diet during childhood and adolescence, as well as bridging symptoms associated with dietary patterns. The results suggest that different intervention methods should be adopted for children and adolescents to improve sleep quality. During childhood, interventions should focus on reducing intake of Fast Food and Snacks, while in adolescence, adopting a Nuts-Aquatic products -Potatoes Diet Type is recommended.

## Data Availability

The original contributions presented in the study are included in the article/Supplementary material, further inquiries can be directed to the corresponding author/s.
