# Supplementary material for: Association between dietary patterns and sleep quality in Chinese children and adolescents: large-scale cross-sectional network analysis
Source: Front Nutr. 2026 Jul 1;13:1851693. doi: 10.3389/fnut.2026.1851693 (PMC13368495; doi:10.3389/fnut.2026.1851693)
Supplement: Supplementary file 1 [file Table_1.DOCX]

Supplementary Material

Table S1. Network Weight Matrix of Children.

|  | **Die1** | **Die2** | **Die3** | **Die4** | **Sleep1** | **Sleep2** | **Sleep3** | **Sleep4** | **Sleep5** | **Sleep6** | **Sleep7** |
| --- | --- | --- | --- | --- | --- | --- | --- | --- | --- | --- | --- |
| Die1 | 0.000 | 0.000 | 0.030 | 0.030 | -0.025 | -0.051 | -0.009 | -0.005 | 0.000 | 0.000 | 0.000 |
| Die2 | 0.000 | 0.000 | 0.000 | 0.000 | -0.013 | -0.039 | -0.031 | -0.019 | 0.000 | -0.054 | 0.000 |
| Die3 | 0.030 | 0.000 | 0.000 | 0.013 | 0.002 | 0.000 | 0.000 | 0.057 | 0.027 | 0.005 | 0.077 |
| Die4 | 0.030 | 0.000 | 0.013 | 0.000 | -0.018 | 0.027 | 0.000 | 0.000 | 0.000 | 0.020 | 0.000 |
| Sleep1 | -0.025 | -0.013 | 0.002 | -0.018 | 0.000 | 0.000 | 0.023 | 0.177 | 0.297 | 0.000 | 0.055 |
| Sleep2 | -0.051 | -0.039 | 0.000 | 0.027 | 0.000 | 0.000 | 0.569 | 0.022 | 0.007 | 0.005 | 0.000 |
| Sleep3 | -0.009 | -0.031 | 0.000 | 0.000 | 0.023 | 0.569 | 0.000 | 0.000 | 0.003 | 0.025 | 0.013 |
| Sleep4 | -0.005 | -0.019 | 0.057 | 0.000 | 0.177 | 0.022 | 0.000 | 0.000 | 0.111 | 0.022 | 0.160 |
| Sleep5 | 0.000 | 0.000 | 0.027 | 0.000 | 0.297 | 0.007 | 0.003 | 0.111 | 0.000 | 0.079 | 0.240 |
| Sleep6 | 0.000 | -0.054 | 0.005 | 0.020 | 0.000 | 0.005 | 0.025 | 0.022 | 0.079 | 0.000 | 0.109 |
| Sleep7 | 0.000 | 0.000 | 0.077 | 0.000 | 0.055 | 0.000 | 0.013 | 0.160 | 0.240 | 0.109 | 0.000 |

*Note*: Die1: High-Protein Diet Type; Die2: Plant-Based Diet Type; Die3: Fast Food and Snack Diet Type; Die4: Nuts-Aquatic products -Potatoes Diet Type; Sleep1: Sleep Quality; Sleep2: Sleep Efficiency; Sleep3: Sleep Duration; Sleep4: Sleep Latency; Sleep5: Daytime Function; Sleep6: Drug Use; Sleep7: Sleep Disturbance

**Table S2**. Network Weight Matrix of Adolescent

|  | **Die1** | **Die2** | **Die3** | **Die4** | **Sleep1** | **Sleep2** | **Sleep3** | **Sleep4** | **Sleep5** | **Sleep6** | **Sleep7** |
| --- | --- | --- | --- | --- | --- | --- | --- | --- | --- | --- | --- |
| Die1 | 0.000 | 0.000 | -0.018 | 0.000 | 0.000 | -0.081 | 0.047 | 0.000 | 0.084 | -0.010 | -0.001 |
| Die2 | 0.000 | 0.000 | 0.000 | 0.000 | -0.031 | -0.060 | -0.049 | -0.027 | 0.000 | -0.046 | 0.000 |
| Die3 | -0.018 | 0.000 | 0.000 | 0.000 | 0.000 | 0.000 | -0.017 | 0.039 | 0.036 | 0.000 | 0.078 |
| Die4 | 0.000 | 0.000 | 0.000 | 0.000 | -0.047 | 0.041 | -0.065 | 0.000 | -0.098 | 0.031 | 0.000 |
| Sleep1 | 0.000 | -0.031 | 0.000 | -0.047 | 0.000 | -0.014 | 0.114 | 0.257 | 0.366 | 0.003 | 0.106 |
| Sleep2 | -0.081 | -0.060 | 0.000 | 0.041 | -0.014 | 0.000 | 0.351 | 0.000 | -0.063 | 0.026 | 0.000 |
| Sleep3 | 0.047 | -0.049 | -0.017 | -0.065 | 0.114 | 0.351 | 0.000 | 0.078 | 0.143 | 0.050 | 0.000 |
| Sleep4 | 0.000 | -0.027 | 0.039 | 0.000 | 0.257 | 0.000 | 0.078 | 0.000 | 0.145 | 0.074 | 0.179 |
| Sleep5 | 0.084 | 0.000 | 0.036 | -0.098 | 0.366 | -0.063 | 0.143 | 0.145 | 0.000 | 0.044 | 0.247 |
| Sleep6 | -0.010 | -0.046 | 0.000 | 0.031 | 0.003 | 0.026 | 0.050 | 0.074 | 0.044 | 0.000 | 0.126 |
| Sleep7 | -0.001 | 0.000 | 0.078 | 0.000 | 0.106 | 0.000 | 0.000 | 0.179 | 0.247 | 0.126 | 0.000 |

*Note*: Die1: High-Protein Diet Type; Die2: Plant-Based Diet Type; Die3: Fast Food and Snack Diet Type; Die4: Nuts-Aquatic products -Potatoes Diet Type; Sleep1: Sleep Quality; Sleep2: Sleep Efficiency; Sleep3: Sleep Duration; Sleep4: Sleep Latency; Sleep5: Daytime Function; Sleep6: Drug Use; Sleep7: Sleep Disturbance


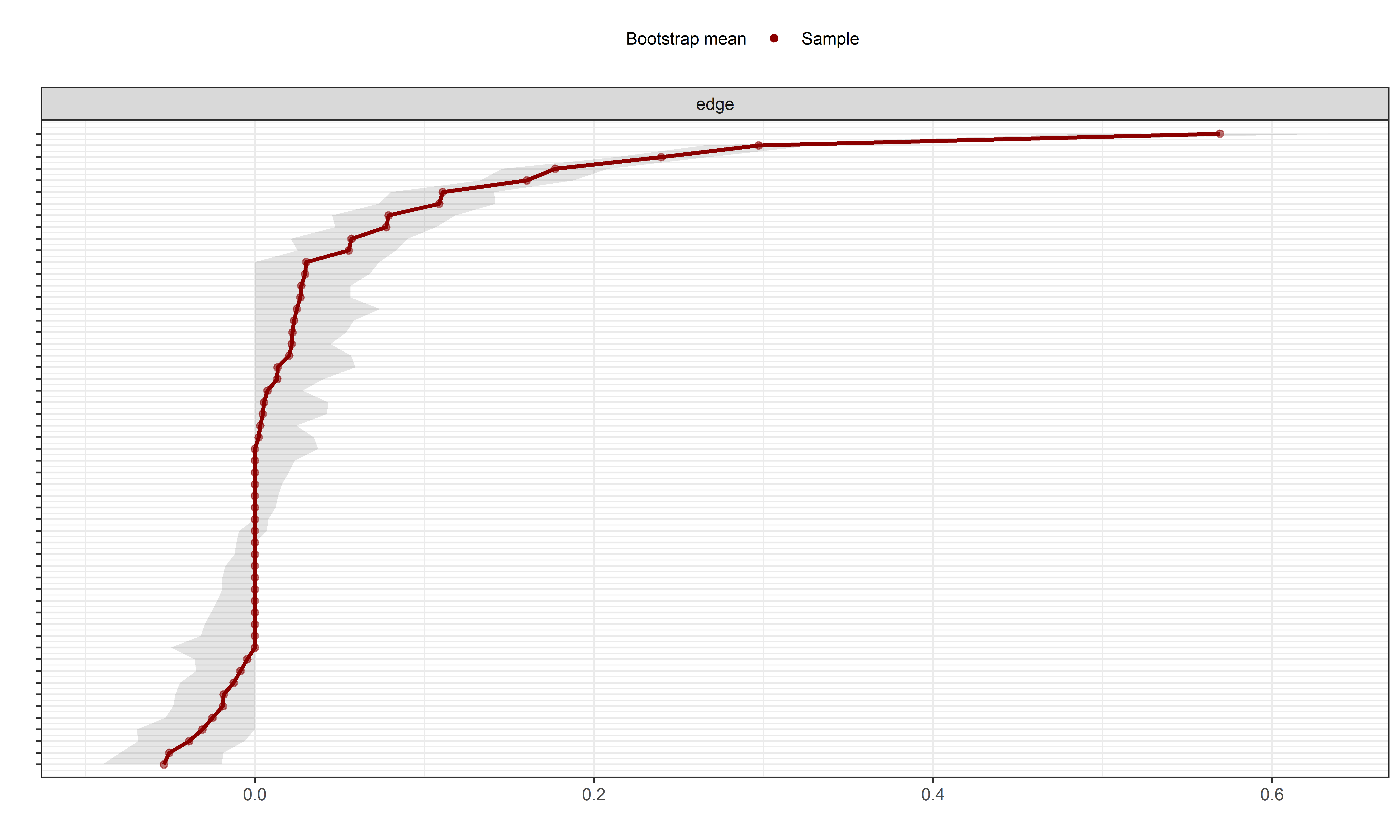


**Figure S1**. Bootstrapped Confidence Intervals of Edge Weights in Children.

*Note*: The black dots indicate the values of each edge weight, ordered from the highest to the lowest value. The gray area represents the 95% Confidence Intervals of edge weights, estimated with the non-parametric bootstrap procedure. Wide intervals indicate lower stability and narrow intervals indicate higher stability


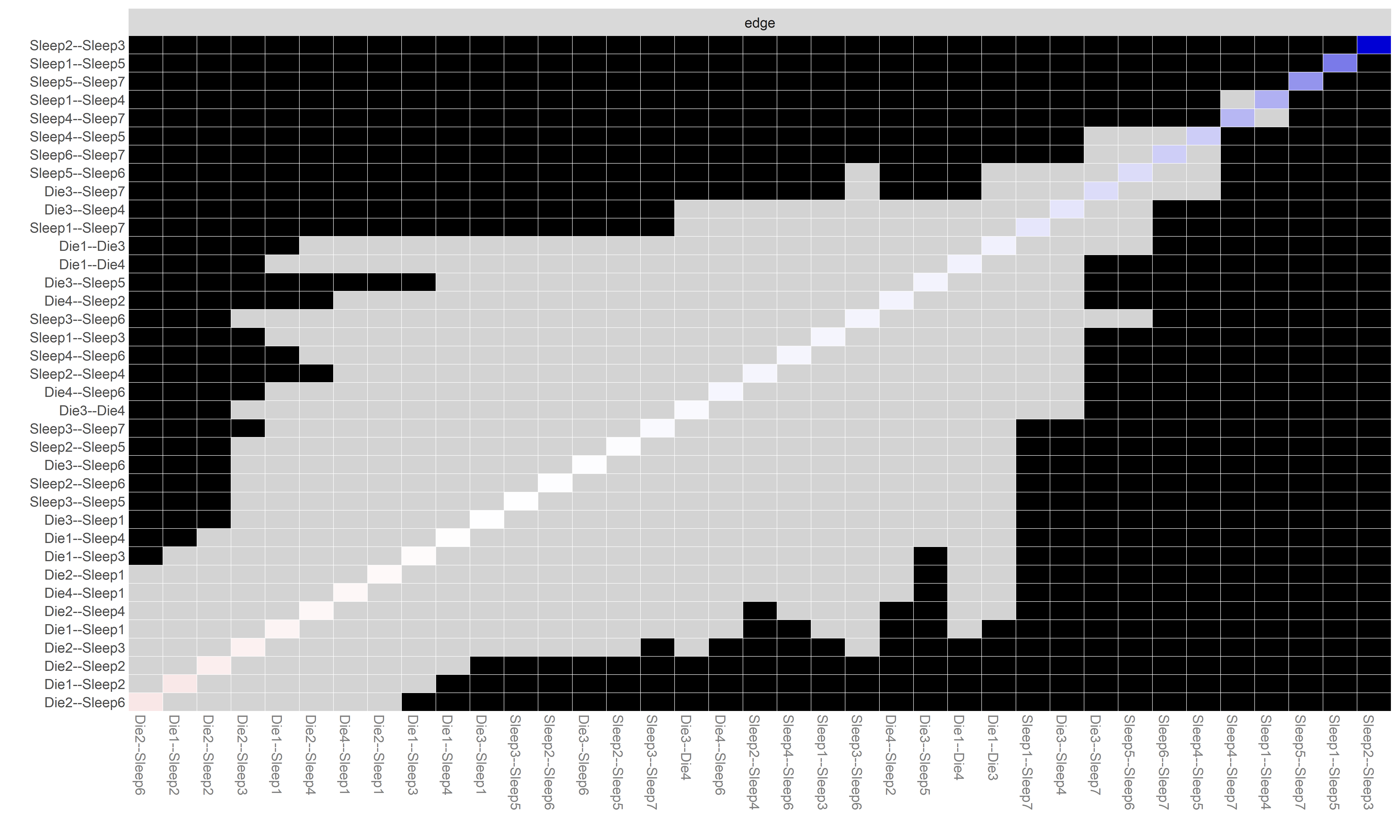


**Figure S2**. Bootstrapped Confidence Intervals of Edge Weights in Children.

*Note*: Bootstrapped difference tests between edge weights in the network. Gray boxes indicate edges that do not significantly differ from one-another. Black boxes represent edges with significant difference from one another (α = 0.05). Blue boxes in the edge-weight plot indicate positive correlations.


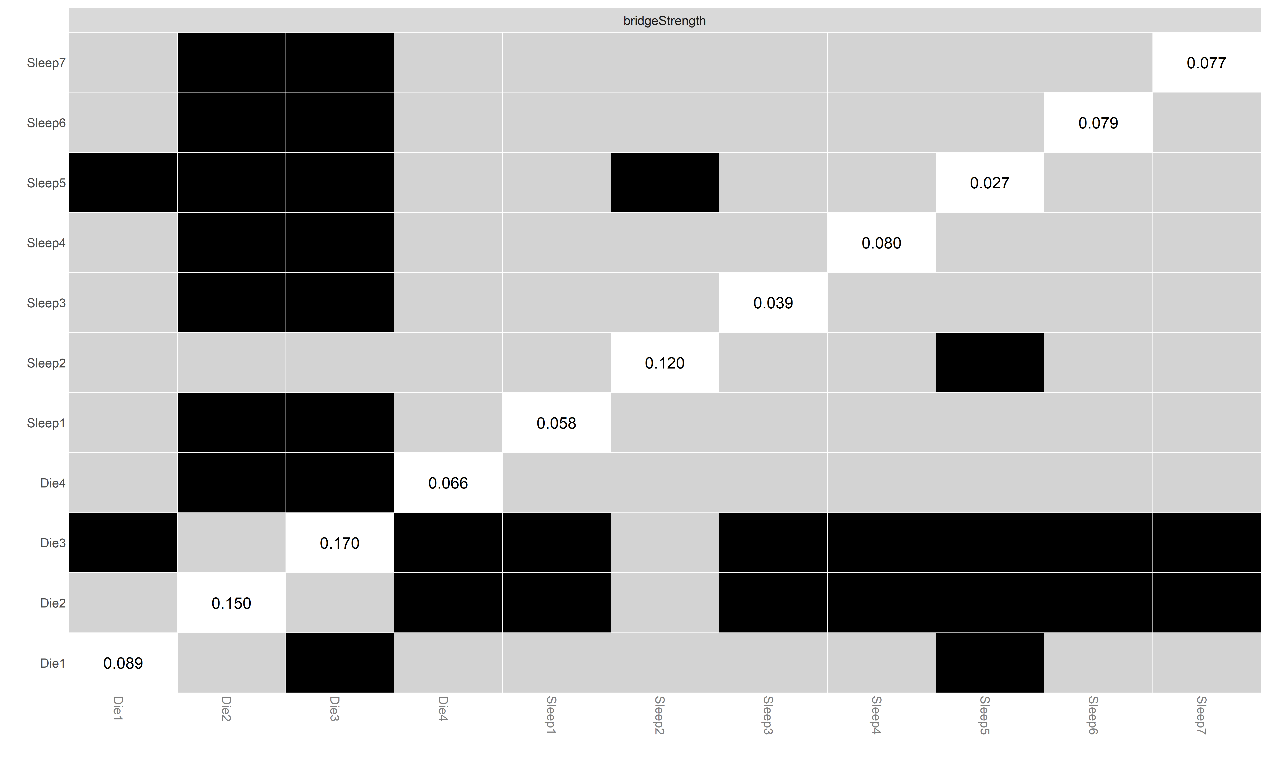


**Figure S3.** Bootstrap Difference Test for Edge Weights in Children.

*Note*: Bootstrapped difference tests between nodes strength in the network. Gray boxes indicate nodes that do not significantly differ from one-another. Black boxes represent nodes that differ significantly from one another (α = 0.05). White boxes show the values of node strength.


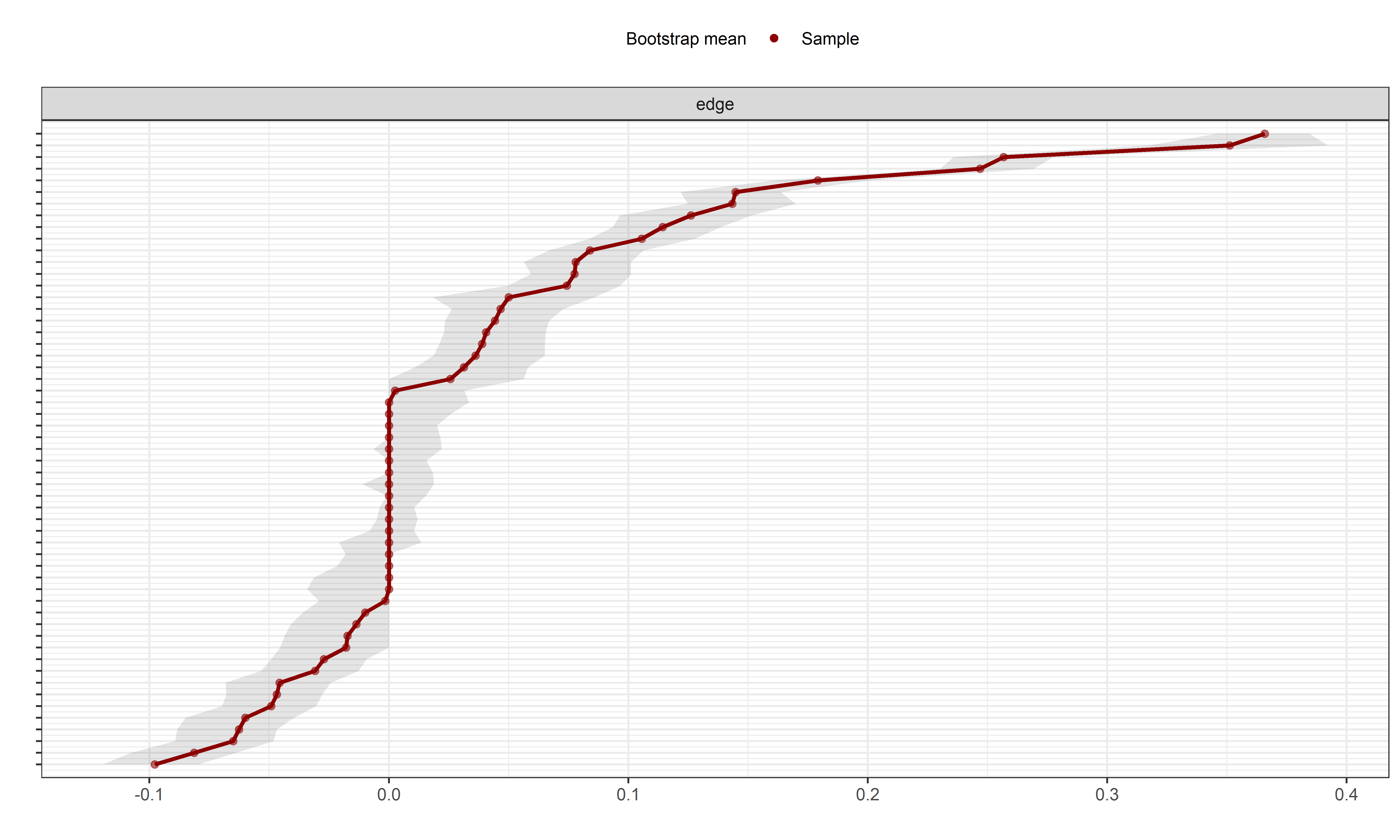


**Figure S4.** Bootstrapped Confidence Intervals of Edge Weights in Adolescents.

*Note*: The black dots indicate the values of each edge weight, ordered from the highest to the lowest value. The gray area represents the 95% Confidence Intervals of edge weights, estimated with the non-parametric bootstrap procedure. Wide intervals indicate lower stability and narrow intervals indicate higher stability.


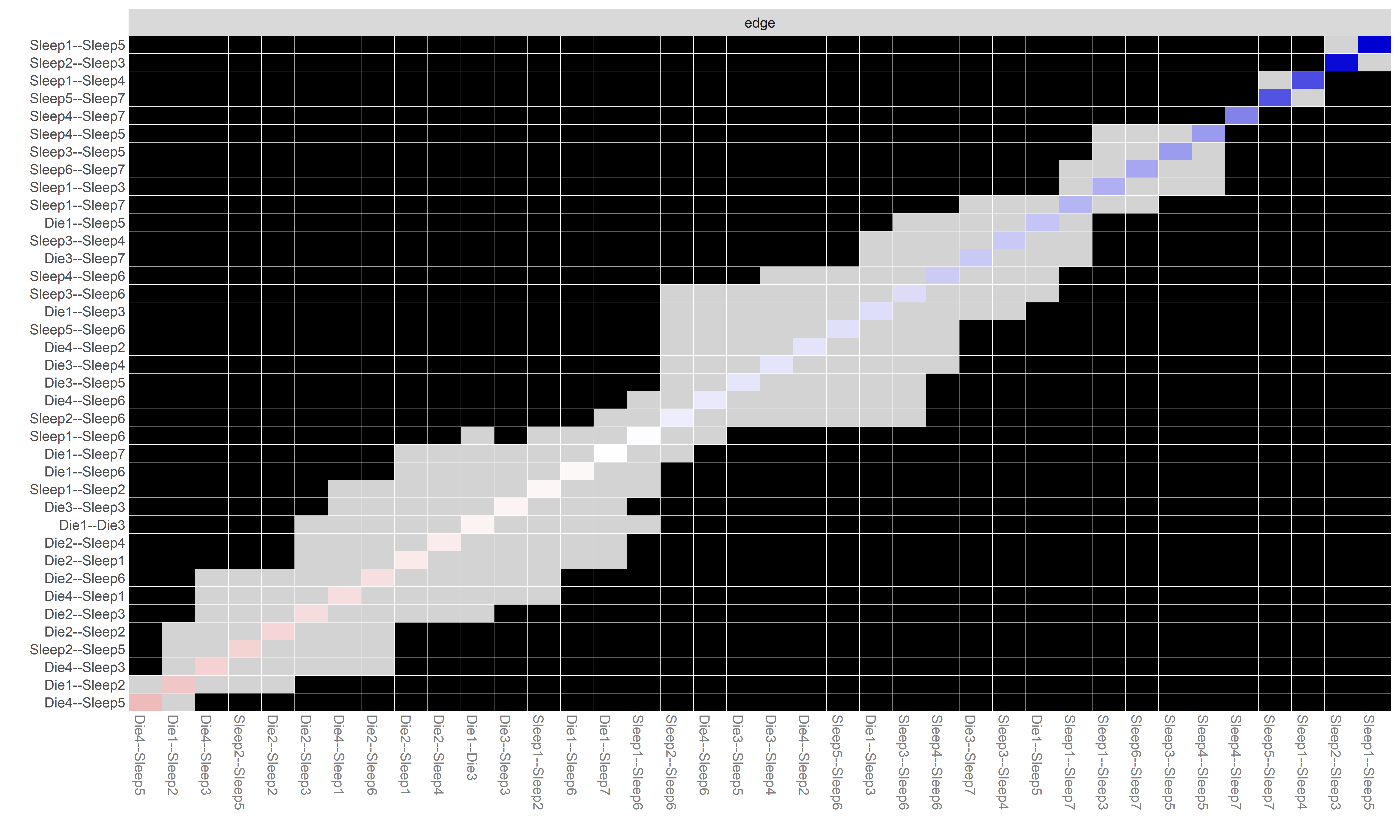


**Figure S5.** Bootstrapped Confidence Intervals of Edge Weights in Adolescents.

*Note*: Bootstrapped difference tests between edge weights in the network. Gray boxes indicate edges that do not significantly differ from one-another. Black boxes represent edges with significant difference from one another (α = 0.05). Blue boxes in the edge-weight plot indicate positive correlations.


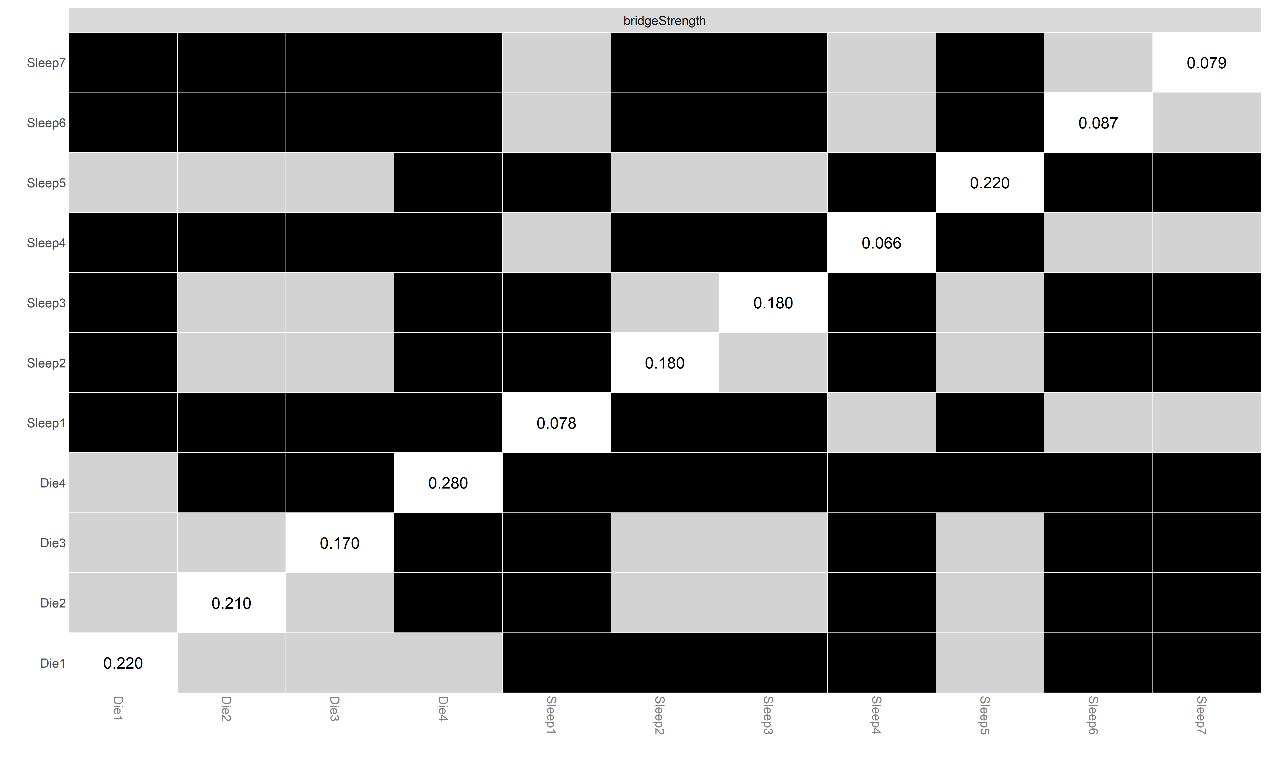


**Figure S6.** Bootstrap Difference Test for Edge Weights in Adolescents. Bootstrapped difference tests between nodes strength in the network. Gray boxes indicate nodes that do not significantly differ from one-another. Black boxes represent nodes that differ significantly from one another (α = 0.05). White boxes show the values of node strength.

**Table S3.** Marginal differences in children and adolescents.

| **Var1** | **Var2** | **p-value** | **statistic E** |
| --- | --- | --- | --- |
| High-Protein Diet Type | Plant-Based Diet Type | 1.000 | 0.000 |
| High-Protein Diet Type | Fast Food and Snack Diet Type | 0.001 | 0.048 |
| Plant-Based Diet Type | Fast Food and Snack Diet Type | 1.000 | 0.000 |
| High-Protein Diet Type | Nuts-Aquatic products -Potatoes  Diet Type | 0.021 | 0.030 |
| Plant-Based Diet Type | Nuts-Aquatic products -Potatoes  Diet Type | 1.000 | 0.000 |
| Fast Food and Snack Diet Type | Nuts-Aquatic products -Potatoes  Diet Type | 0.128 | 0.013 |
| **Nuts-Aquatic products -Potatoes**  **Diet Type** | **Sleep Duration** | **0.003** | **0.065** |
| Plant-Based Diet Type | Sleep Quality | 0.306 | 0.018 |
| Fast Food and Snack Diet Type | Sleep Quality | 0.204 | 0.002 |
| Nuts-Aquatic products -Potatoes  Diet Type | Sleep Quality | 0.106 | 0.028 |
| High-Protein Diet Type | Sleep Efficiency | 0.166 | 0.031 |
| Plant-Based Diet Type | Sleep Efficiency | 0.277 | 0.021 |
| Fast Food and Snack Diet Type | Sleep Efficiency | 1.000 | 0.000 |
| Nuts-Aquatic products -Potatoes  Diet Type | Sleep Efficiency | 0.532 | 0.014 |
| Sleep Quality | Sleep Efficiency | 0.546 | 0.014 |
| **Nuts-Aquatic products -Potatoes**  **Diet Type** | **Daytime function** | **0.001** | **0.098** |
| Plant-Based Diet Type | Sleep Duration | 0.318 | 0.018 |
| Fast Food and Snack Diet Type | Sleep Duration | 0.443 | 0.017 |
| **High-Protein Diet Type** | **Sleep Quality** | **0.004** | **0.025** |
| Sleep Quality | Sleep Duration | 0.001 | 0.091 |
| Sleep Efficiency | Sleep Duration | 0.001 | 0.218 |
| High-Protein Diet Type | Sleep Latency | 0.067 | 0.005 |
| Plant-Based Diet Type | Sleep Latency | 0.640 | 0.008 |
| Fast Food and Snack Diet Type | Sleep Latency | 0.354 | 0.018 |
| Nuts-Aquatic products -Potatoes  Diet Type | Sleep Latency | 1.000 | 0.000 |
| Sleep Quality | Sleep Latency | 0.001 | 0.080 |
| Sleep Efficiency | Sleep Latency | 0.004 | 0.022 |
| Sleep Duration | Sleep Latency | 0.002 | 0.078 |
| **High-Protein Diet Type** | **Sleep Duration** | **0.014** | **0.055** |
| Plant-Based Diet Type | Daytime function | 1.000 | 0.000 |
| Fast Food and Snack Diet Type | Daytime function | 0.624 | 0.009 |
| **High-Protein Diet Type** | **Daytime function** | **0.001** | **0.084** |
| Sleep Quality | Daytime function | 0.001 | 0.069 |
| Sleep Efficiency | Daytime function | 0.001 | 0.070 |
| Sleep Duration | Daytime function | 0.001 | 0.140 |
| Sleep Latency | Daytime function | 0.082 | 0.034 |
| High-Protein Diet Type | Drug use | 0.638 | 0.010 |
| Plant-Based Diet Type | Drug use | 0.672 | 0.008 |
| Fast Food and Snack Diet Type | Drug use | 0.372 | 0.005 |
| Nuts-Aquatic products -Potatoes  Diet Type | Drug use | 0.641 | 0.011 |
| Sleep Quality | Drug use | 0.232 | 0.003 |
| Sleep Efficiency | Drug use | 0.445 | 0.021 |
| Sleep Duration | Drug use | 0.419 | 0.025 |
| Sleep Latency | Drug use | 0.013 | 0.052 |
| Daytime function | Drug use | 0.068 | 0.035 |
| High-Protein Diet Type | Sleep disturbance | 0.369 | 0.001 |
| Plant-Based Diet Type | Sleep disturbance | 1.000 | 0.000 |
| Fast Food and Snack Diet Type | Sleep disturbance | 0.999 | 0.000 |
| Nuts-Aquatic products -Potatoes  Diet Type | Sleep disturbance | 1.000 | 0.000 |
| Sleep Quality | Sleep disturbance | 0.007 | 0.050 |
| Sleep Efficiency | Sleep disturbance | 1.000 | 0.000 |
| Sleep Duration | Sleep disturbance | 0.022 | 0.013 |
| Sleep Latency | Sleep disturbance | 0.276 | 0.019 |
| Daytime function | Sleep disturbance | 0.638 | 0.007 |
| Drug use | Sleep disturbance | 0.489 | 0.017 |
